# Ultra-Miniature Circularly Polarized CPW-Fed Implantable Antenna Design and its Validation for Biotelemetry Applications

**DOI:** 10.1038/s41598-020-63780-4

**Published:** 2020-04-22

**Authors:** Vikrant Kaim, Binod Kumar Kanaujia, Sachin Kumar, Hyun Chul Choi, Kang Wook Kim, Karumudi Rambabu

**Affiliations:** 1 School of Computational and Integrative Sciences, Jawaharlal Nehru University, New Delhi, 110067 India; 2 School of Electronics Engineering, Kyungpook National University, Daegu, 41566 Republic of Korea; 3 Department of Electrical and Computer Engineering, University of Alberta, Edmonton, Alberta, T6G 2V4 Canada

**Keywords:** Biomedical engineering, Electrical and electronic engineering

## Abstract

The paper presents a coplanar waveguide (CPW)-fed ultra-miniaturized patch antenna operating in Industrial, Scientific and Medical (ISM) band (2.4–2.5 GHz) for biotelemetry applications. The proposed antenna structure is circular in shape and its ground plane is loaded with a pair of slots for obtaining circular polarization. In the proposed design, asymmetric square slots generate phase condition for right-hand circularly polarized (RHCP) radiation. And, by merely changing the position of the slots, either RHCP or left-hand circularly polarized (LHCP) radiation can be excited. In the proposed design, a meandered central strip is used for miniaturization. The simulations of the proposed antenna are carried out using Ansys HFSS software with a single-layer and multilayer human tissue models. The antenna shows good performance for different tissue properties owing to its wide axial ratio bandwidth and impedance bandwidth. The antenna is fabricated and measurements are carried out in skin mimicking phantom and pork. Simulated and measured performances of the antenna are in close agreement. The power link budget is also calculated using an exterior circularly polarized (CP) receiving antenna.

## Introduction

Recent advances in technology lead to the design of small and low-power consuming biomedical devices that can be implanted inside a patient’s body through surgical operation or ingestion. These embedded devices can sense data from inside the human body in real-time, offering a unique opportunity for early diagnosis and treatment of diseases. The embedded devices communicate with the external world in terms of telemetry. Telemetry includes data transmission from the implanted device to an external one and vice-versa.

The standard requirement of all implantable medical devices (IMD) is the wireless operation of equipment and bidirectional data communication. The implantable antenna is one of the critical components for IMD for exchanging body anatomy data with installed base stations^1,2^. In the last few years, several prototypes of implantable patch antennas are proposed; but their radiation efficiency lacks in one or the other parameters such as gain, impedance bandwidth, axial ratio bandwidth; in few cases, antenna footprints are also large for implantation^3–11^. In^12–16^, dimensions of the antenna are significantly reduced making them best prototypes for implantation, but are prone to multipath fading because of their omnidirectional linearly polarized radiations. Though these antenna designs are highly compact, their gain, and impedance bandwidths are very less. Various types of antennas with a defected ground, fractal shape, spiral, slotted, PIFA have been proposed in the literature for wide impedance and axial ratio bandwidth^17–20^. However, most of the proposed antennas are linearly polarized with large and complex geometry. Few antennas reported consist of ground plane as the primary radiator, but most of them are linearly polarized and proposed for wireless applications. Since the profile of such antennas is relatively low, due to the involvement of a single metal layer, so suits for the implantable applications^21–26^. Hence, for biomedical applications, the primary requirement is a small physical size with excellent radiation characteristics, which can be fulfilled using ground radiating antennas.

In order to reduce multipath fading effects and improve data rate, circularly polarized (CP) antennas are recommended. A CP helical antenna for ingestible application was presented in^27^. The reported helical antenna has a high profile due to its multilayer structure making it inconvenient for implantable applications. Another design of a capacitively-loaded CP antenna was presented for Industrial, Scientific and Medical (ISM) band biomedical applications. However, its axial ratio bandwidth is narrow and is sensitive to the properties of human tissues^28^. In^29^, a patch loaded radiated loop CP antenna using slow-wave concept was reported for ISM band, but its gain and bandwidth are very less. Stub loaded CP implantable antenna was presented in^30^. For size reduction interdigital capacitive coupling was employed but still, the antenna footprints are not compatible for body implantation. The impedance and axial ratio bandwidths were narrow with very less gain. Another CP implantable annular ring antenna was presented in^31^. CP radiation is generated by using open stubs in the annular ring. Z-shaped slot improves axial ratio bandwidth, but the overall radiation performance lacks far behind. A ground radiating antenna with lumped components was reported in^32^. Although, the antenna is highly miniaturized but has low gain and narrow axial ratio bandwidth, and the use of reactive components makes the fabrication process complicated. Hence, for implantable applications, there is a need for a low profile, compact size antenna with wide impedance and axial ratio bandwidths, and high gain in the ISM band. The IEEE 802.15.4 standard 2.4–2.5 GHz band is adapted in this paper as it is universal, interoperable with high gain.

In this paper, a circular-shaped ground radiating patch antenna operating in ISM (2.4–2.5 GHz) band is proposed for biomedical implantable applications. A circular configuration is selected to have a comparable antenna shape and size with medical tablets (pills) avoiding sharp corners facilitating ingestion and implantable applications. The main focus of the paper is designing a miniaturized antenna structure with acceptable performance and validating the antenna for biotelemetry applications. By embedding asymmetric square slots in the ground with respect to coplanar waveguide (CPW) feed, CP radiation property is obtained by realizing a quadrature-phase difference. Initially, the proposed antenna was designed and analyzed using homogeneous simplified single and multilayer human tissue models consisting of skin, fat and muscle tissues through FEM-based Ansys HFSS (v 19.2). Later, the proposed antenna performance was validated within the heterogeneous environment of the head, chest, hand, liver, stomach and small intestine of the AustinMan (v 2.6) voxel model of the human body using CST Microwave Studio Suite. Further, antenna parameters are measured in skin mimicking phantom and pork, and compared with the results of simulator Ansys HFSS. In Table 1, the results of the proposed antenna are compared with the work reported in the literature. Finally, specific absorption rate (SAR) is calculated using CST Studio Suite in addition to link budget calculations.

**Table 1:** Comparison of the proposed antenna with other reported antennas.

Ref.	8	9	10	11	12	13	14	15	16	27	28	29	30	31	32	Prop. work
Antenna type	PIFA	Hybrid patch/slot	Slot	PIFA	Meander patch	PIFA	Flower shape patch	Ring slot	Planar folded meander dipole	Helical	Capacitive load patch	Loop	Stub loaded patch	Annular ring	Ground radiation	Circular ground radiation
Central freq. (GHz)	0.402, 2.45	0.402	0.402	0.403	0.915, 2.45	0.915, 1.9, 2.45	0.928, 2.45	2.45	0.402	2.45	2.45	0.915	0.915	2.45	2.45	2.45
**Freq. band**	**MICS, ISM**	**MICS**	**MICS**	**Med-radio**	**ISM**	**ISM, Midfield**	**ISM**	**ISM**	**Med-radio**	**ISM**	**ISM**	**ISM**	**ISM**	**ISM**	**ISM**	**ISM**
Area (mm^2^)	100	160	110	156.25	48	42	50.4	100	66.88	133 (2π*rh*)	100	169	121	95	108.16	85 (π*r*^2^)
Volume (mm^3^)	245	203.2	139.7	198.4	24	21	10.08	40	20	367 (π*r*^2^*h*)	127	214.63	154	121	54.94	43.13 (π*r*^2^*h*)
**Simulation enclosure**	**Skin**	**Skin**	**Skin**	**Muscle**	**Skin**	**Skin**	**Skin**	**Muscle**	**Skin, muscle, bone**	**Muscle**	**Skin**	**Skin**	**Skin**	**Skin**	**Skin**	**Skin**
Enclosure size (mm^3^)	65 × 92 × 50	96 × 90 × 7.27	60 × 180 × 60	92.5 × 92.5 × 39.27	200 × 200 × 200	200 × 200 × 200	100 × 100 × 100	80 × 80 × 80	100 × 70 × 70	100 × 100 × 100	90 × 90 × 25.27	60 × 180 × 60	100 × 100 × 50	90.4 × 90.4 × 25.27	120 × 120 × 75	120 × 120 × 75
Implant depth (mm)	—	3	3	5	4	4.5	50	40	2	50	4	3	4	4	4	4
Impedance BW (%)	28, 3	22.8	28.3	7.26	9.84, 8.57	8.7, 8.2, 7.3	19.8, 8.96	57	35	26	10.2	27.8	4	8.3	25.3	99.25
Axial ratio BW (%)	—	—	—	—	—	—	—	—	—	33.3	1.63	18.2	1.2	2.49	10.2	63.56
Peak gain (dBc)	−7, −15	−30.5	−27.7	−32.49	−28.5, −22.8	−26.4, −23, −21	−28.74, −25.65	−9	−23.7	−32	−22	−32	−29	−22.7	−21.1	−15
Isolation (dB)	—	—	—	—	—	—	—	—	—	27	22	—	20	—	30	25

### Antenna geometry and design

**Figure 1:**
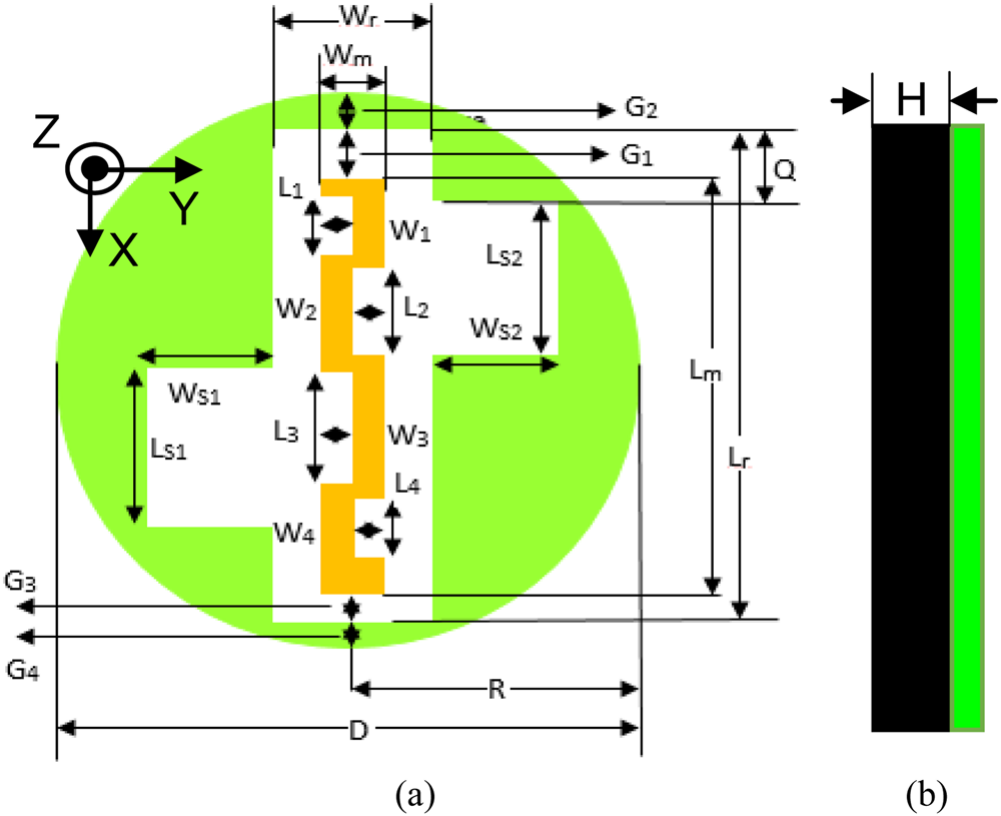
Schematic of the proposed ground radiating antenna (**a**) top view (**b**) side view.

**Figure 2:**
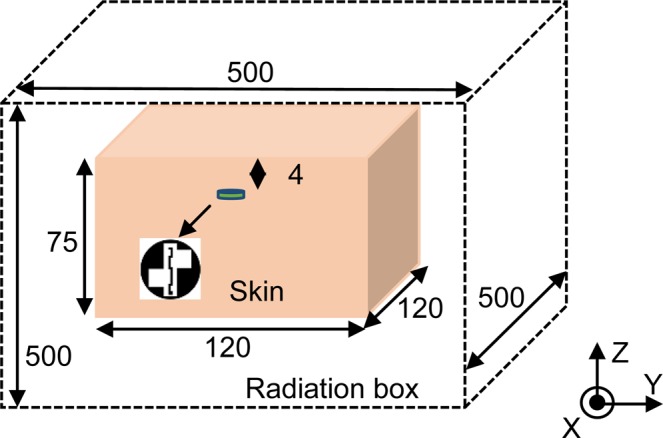
Schematic of the simulation enclosure.

Figure 1(a),(b) shows the geometry of the proposed circular ground radiating CPW-fed antenna. A vertical rectangular slot and two square slots are etched from the ground plane of the antenna to achieve circular polarization and compactness. As can be seen in Fig. 1(a), the antenna is excited using a coplanar feed at *G*_3_. A gap of 0.3 mm is maintained (at feeding point) between the meandered central patch strip and ground plane for impedance matching. The antenna is fabricated on RT Duroid 5880 substrate (*ɛ*_*r*_ = 2.2, tan *δ* = 0.0009) of thickness (*H*) 0.508 mm. The detailed design parameter values are given in Table 2. The proposed antenna is placed in the middle of a homogeneous simplified one-layer human skin tissue model to conduct simulations, locating 4 mm below the top surface, as shown in Fig. 2. The dimensions of the tissue model are 120 × 120 × 75 mm^3^. This model matches to the electrical properties of the skin (at 2.45 GHz), having a relative dielectric constant of 37.88 and conductivity of 1.44 S/m^32^. The radiation boundaries are kept at a distance greater than *λ*_o_/4 at 2.45 GHz. The dimensions in the paper are in millimeter (mm) unless stated.

**Table 2:** Design parameters of the proposed design. (in mm).

*D*	10.4	*L*_*S*1_	3.15	*L*_2_	1.5	*G*_1_	1
*R*	5.2	*W*_*S*1_	2.45	*W*_2_	0.55	*G*_2_	0.6
*L*_*r*_	9.5	*L*_*S*2_	3.15	*L*_3_	2	*G*_3_	0.3
*W*_*r*_	2.3	*W*_*S*2_	2.45	*W*_3_	0.55	*G*_4_	0.3
*L*_*m*_	8.2	*L*_1_	1.2	*L*_4_	1.2	*Q*	1.6
*W*_*m*_	1.1	*W*_1_	0.55	*W*_4_	0.55

**Figure 3:**
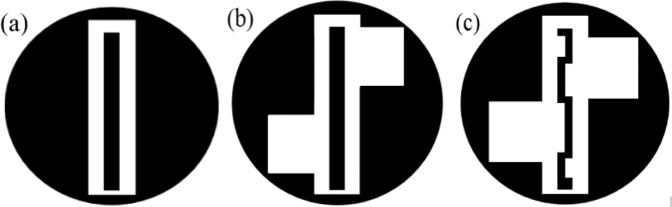
Evolution of the proposed antenna (**a**) Antenna-0 (**b**) Antenna-1 (**c**) Antenna-2.

**Figure 4:**
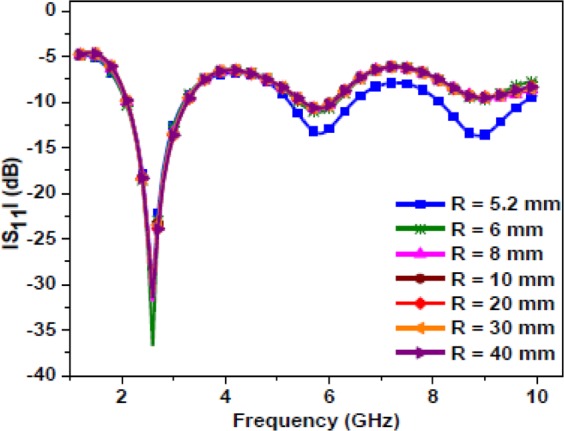
Simulated |S_11_ | plot for Antenna-2 as a function of radius.

**Figure 5:**
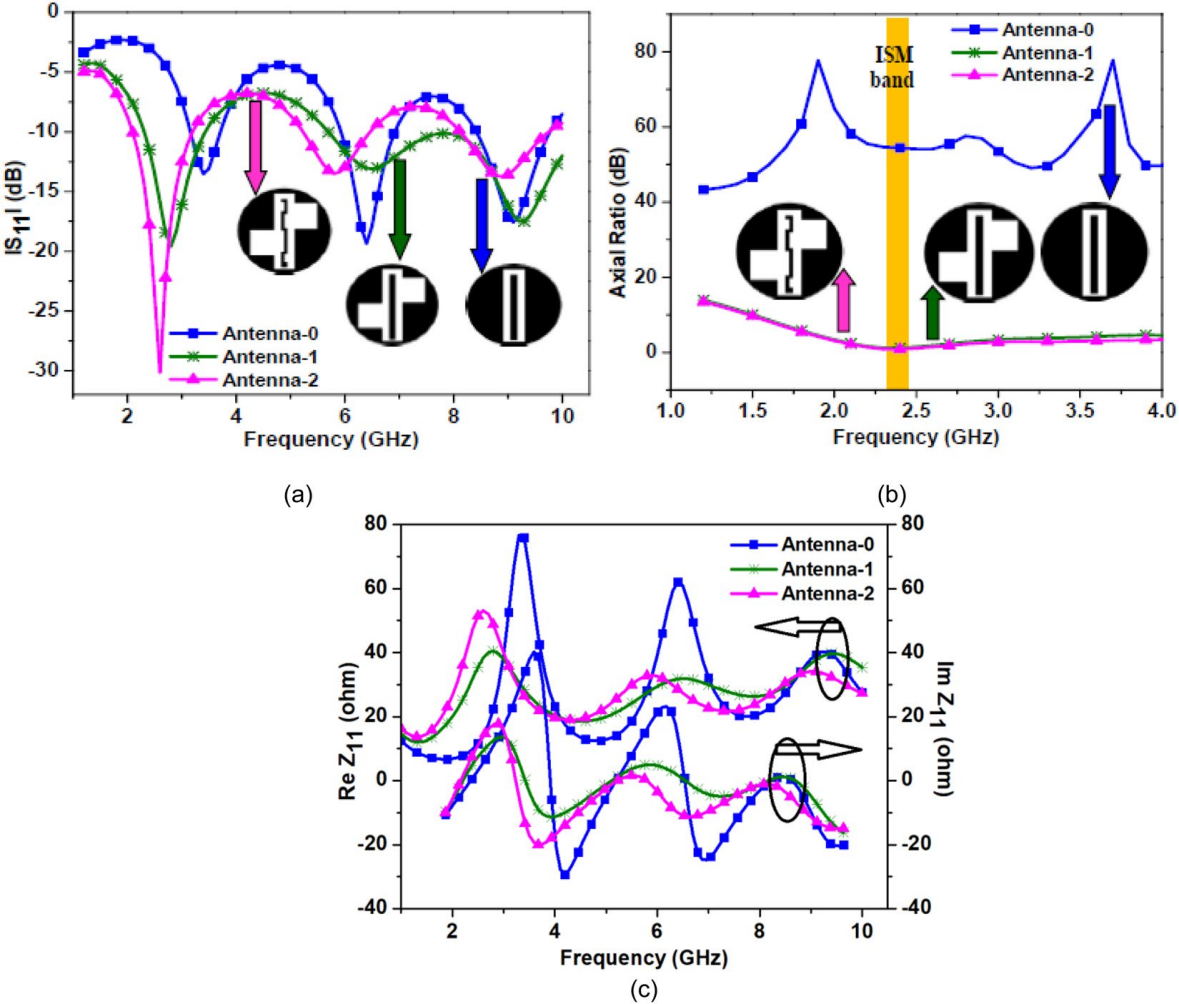
Simulated results for the Antenna-0, Antenna-1, and Antenna-2 (**a**) |S_11_ | (**b**) axial ratio (**c**) input impedance.

Figure 3 illustrates the evolution steps of the proposed antenna design. To have a comparable antenna size with a medical tablet (pill), the value of *R* must be 5.2 mm, or less. In this design, the radius of the antenna doesn’t show a significant role on the resonant frequency, which can be seen in Fig. 4. In the subsequent sub-sections, this concept is explained in detail. Figure 4 shows the simulated |S_11_ | as a function of radius for the proposed Antenna-2 (shown in Fig. 3(c)). Figure 5(a–c) show the simulated |S_11_ | , axial-ratio, and input impedance for the three antennas (Antenna-0, Antenna-1, Antenna-2), respectively. It can be seen from Fig. 5 that Antenna-0, Antenna-1, and Antenna-2 shows resonance at three frequency bands. Also, Antenna-1 and Antenna-2 exhibits circular polarization in their first resonating band.

### Operational mechanism of the antenna

The proposed antenna design evolution begins with the design of Antenna-0 of radius 5.2 mm, which is a typical medical tablet (pill) radius. A vertical rectangular slot of size 9.5 mm × 2.3 mm is etched out from the antenna surface. A metal strip of size 8.2 mm × 1.1 mm is integrated at the center of the rectangular slot (shown in Fig. 3(a)) and this central patch strip is excited by using a coplanar feed. As shown in Fig. 5(a), Antenna-0 shows resonance at frequencies 3.4 GHz (first resonance), 6.4 GHz (second resonance) and 9.1 GHz (third resonance), which are harmonically related.

**Figure 6:**
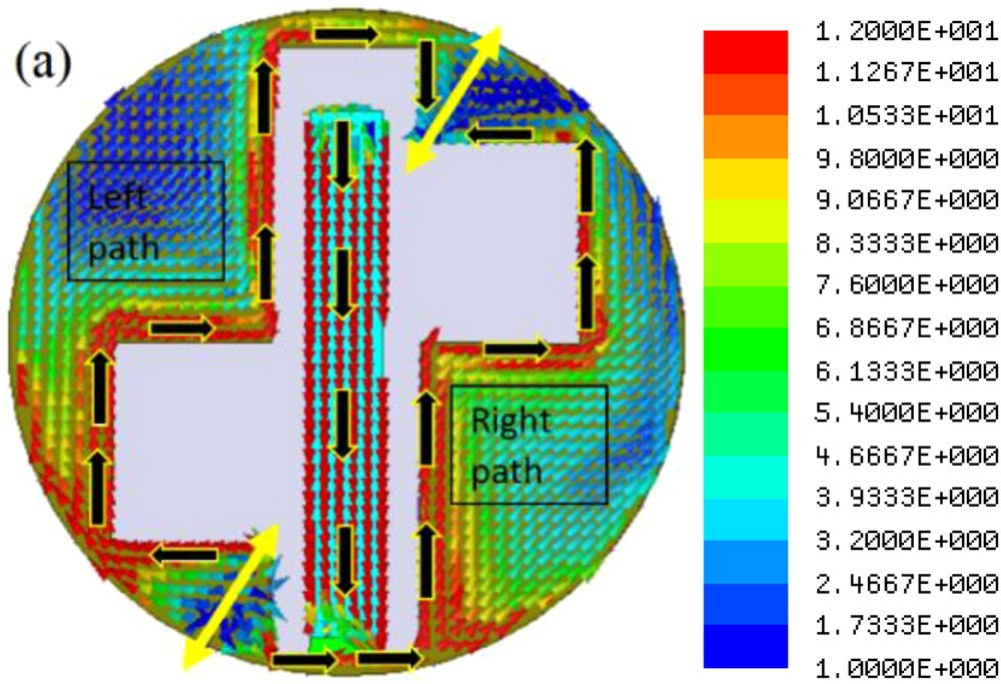
Surface current distribution of the Antenna-1 at 2.8 GHz (scale: Jsurf, A/m).

To achieve circular polarization and miniaturization in the antenna design, Antenna-1 is proposed. Here, as shown in Fig. 3(b), the length of the current path (around the rectangular slot edges) is increased by etching two asymmetrical square slots of size 3.15 mm × 2.45 mm in the ground plane. The Antenna-1 is resonating at frequencies 2.8 GHz (first resonance), 6.5 GHz (second resonance), and 9.3 GHz (third resonance) with −10 dB impedance bandwidth of 40.14%, 33.84%, and 27.95%, respectively. The proposed structure exhibits circular polarization in the first resonating band with 3-dB axial ratio bandwidth of 36.25%. The surface current distribution of Antenna-1 at first resonant frequency is shown in Fig. 6. The second and third resonances (shown in Fig. 5(a)) are essentially the harmonics of the first resonance, hence, the discussion here is restricted to the first resonance frequency only.

### First Resonance (2.8 GHz)

**Figure 7:**
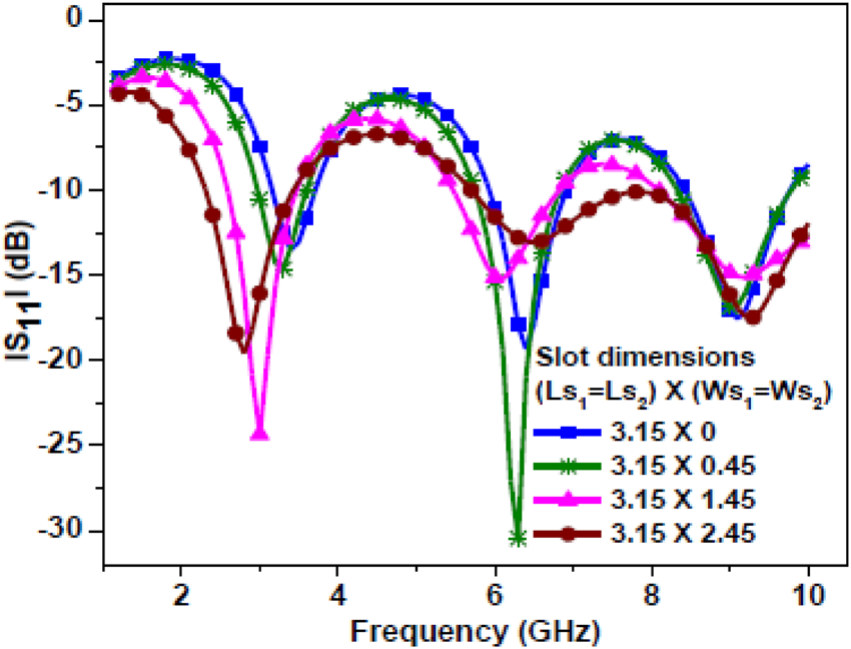
Simulated |S_11_ | plot of the Antenna-1 for different square slot dimensions.

The first resonance is excited due to the combined length of the central patch strip and half of the total slot length. The entire slot length comprises the rectangular slot and the square slots. As shown in Fig. 6, the current distribution is mainly concentrated on the patch strip and around the slot edges. Here, due to the etching of two square slots, asymmetricity with respect to CPW feed arises. Due to this asymmetricity, two continuous current path lengths are formed, namely the right side path and left side path. The right side path comprises the current path length on the central patch strip and half of the total slot length between two yellow lines. Whereas, the left side current path comprises the path length around half of the entire slot length between two yellow lines. Therefore, at this resonance, the current path length (due to the right side path) would be a quarter of the wavelength in the medium. Figure 7 shows a parametric study of the Antenna-1 by varying the square slot dimensions and keeping other dimensions fixed. Thus, the resonating length responsible for first resonance can be calculated as

1 $${R}_{L1}={L}_{m}+{W}_{r}+({L}_{r}-{L}_{L2}-{\rm{Q}})+{W}_{S2}+{L}_{S2}+{W}_{S2}$$

**Figure 8:**
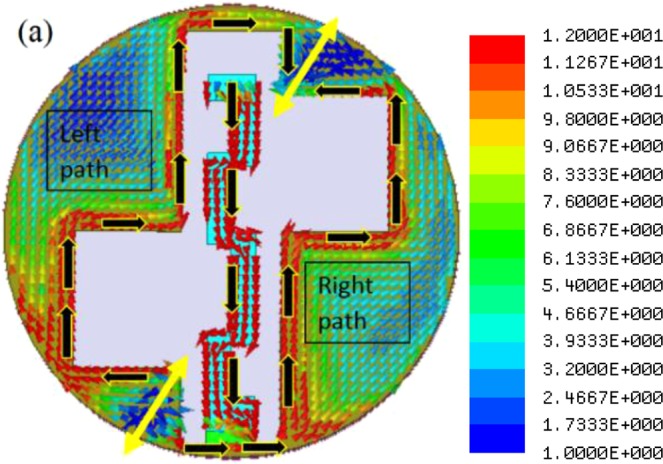
Surface current distribution of the Antenna-2 at 2.6 GHz (scale: Jsurf, A/m).

The resonant frequency can be approximately calculated by using Eq. (1). The ${R}_{L1}$ must be *λ*_*g*_/4 and in the case of Antenna-1, it is 23.3 mm. To achieve further miniaturization and to match the central frequency of the first resonance with ISM band, the Antenna-2 is designed. Here, the path length of the current due to the strip and slot is increased by meandering the central strip (see Fig. 3(c)). The rectangular slot and the two asymmetrical square slots remain unaltered in the proposed design. The Antenna-2 is resonating at frequencies 2.6 GHz (first resonance), 5.8 GHz (second resonance) and 8.9 GHz (third resonance) with −10 dB impedance bandwidth of 42.30%, 22.41% and 19.10%, respectively. The antenna exhibits circular polarization only in the first resonant band with 3-dB axial ratio bandwidth of 61.22%. The surface current distribution of the proposed antenna at first resonant frequency is shown in Fig. 8.

### First resonance (2.6 GHz)

**Figure 9:**
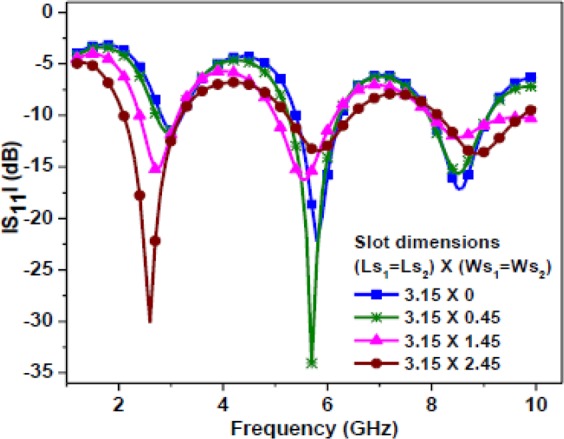
Simulated |S_11_ | plot of the Antenna-2 for different square slot dimensions.

It is observed that a strong current distribution is mainly concentrated on the meandered strip and around the slot edges. Due to asymmetricity, two continuous current path lengths are formed, namely the right side path and left side path as similar to the case of first resonance in Antenna-1. Figure 9 shows a parametric study of the Antenna-2 by varying the square slot dimensions and keeping other dimensions fixed. The resonating length can be calculated as shown in Eq. (1). However, here ${L}_{m}$ is equal to 13.7 mm due to meandering.

### Operation of the antenna for circular polarization

In this section, circular polarization operation and radiation performance of the proposed antenna in the biological environment of the human body has been explained (at the resonating frequency of 2.45 GHz).

**Figure 10:**
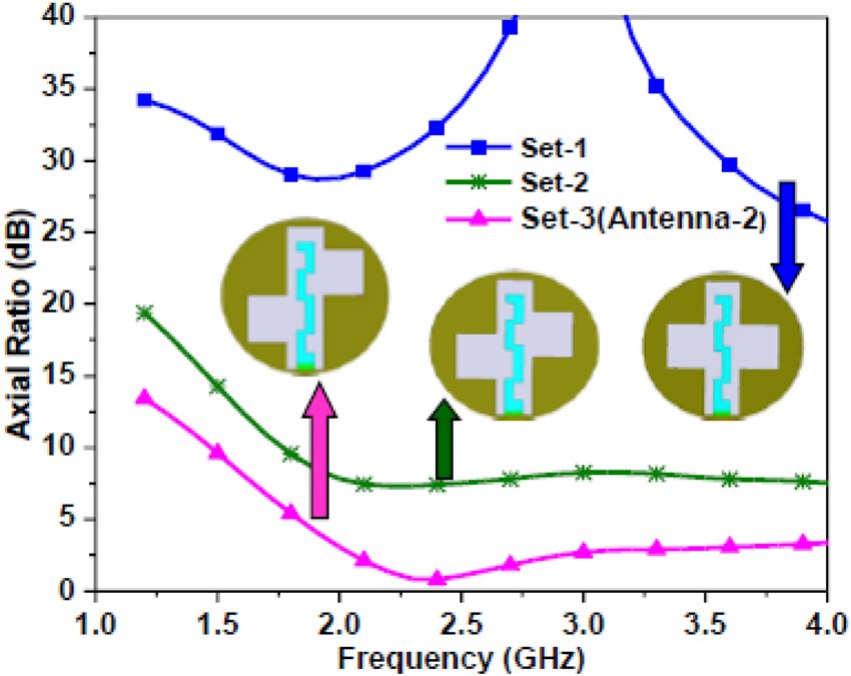
Simulated axial ratio plots of the Antenna-2 for different positions of the square slots.

**Figure 11:**
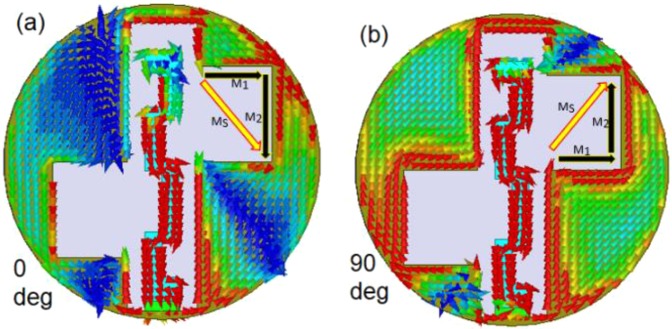
Surface current distributions of the Antenna-2 at 2.45 GHz (**a**) *ωt* = 0° (**b**) *ωt* = 90°.

**Figure 12:**
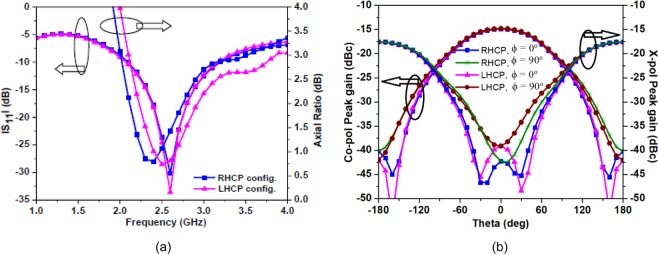
(**a**) Simulated |S_11_ | and axial ratio of the Antenna-2 (for RHCP and LHCP configuration) (**b**) simulated gain patterns at 2.45 GHz of the Antenna-2 (for RHCP and LHCP configuration at *ϕ* = 0° and 90°).

In Antenna-1 and Antenna-2, the asymmetric square slot configurations are designed to generate CP radiation at 2.45 GHz. The square slots make the fundamental resonant mode to split into two near-degenerate orthogonal modes. The position of loaded square slots (with respect to each other) induces phase quadrature between the two orthogonal modes. The axial ratio plots as a function of the position of two square slots for the proposed Antenna-2 are shown in Fig. 10. The positions of two square slots are shifted in set-2 and set-3 from the reference set-1 by shifting the center point of the square slots in the opposite directions by half of ${L}_{S1}/{L}_{S2}$ keeping the dimensions of square slots fixed. To further visualize the CP operation, the surface current distributions are plotted with respect to time phase *ωt* = 0° and *ωt* = 90° as shown in Fig. 11. In Fig. 11, the notations *M*_1_ and *M*_2_ represents orthogonal current vectors, while *M*_*s*_ represents the vector sum of orthogonal current vectors. At *ωt* = 0°, the current density on ${W}_{S2}$ and ${L}_{S2}$ edges of the square slot rises and vector sum points to the lower right side. At *ωt* = 90°, the vector sum points to the upper right side. This vector sum is orthogonal to that at *ωt* = 0° and rotates anti-clockwise as the time progresses, thus, producing right-hand circularly polarized (RHCP) radiation in the $z > 0$ direction. By interchanging the position of the two square slots either RHCP radiation or left-hand circularly polarized (LHCP) radiation can be obtained in the $z > 0$ direction. Figure 12(a) shows the |S_11_ | and axial ratio plot for both RHCP and LHCP configurations of the proposed antenna.

For both the configurations, the proposed antenna performs well such that the broadband performance stays intact in both impedance and axial-ratio bandwidth. Figure 12(b) shows the simulated gain pattern at 2.45 GHz for both the configurations where peak gain at the boresight is −15 dBc, therefore the proposed antenna can work efficiently to handle the menaces of polarization diversity.

### Evaluation of the antenna performance *in Situ*

**Figure 13:**
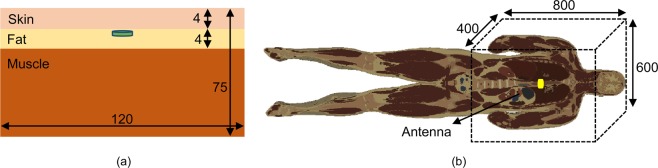
(**a**) Simplified homogeneous three-layer model (**b**) heterogeneous realistic body model (AustinMan).

**Figure 14:**
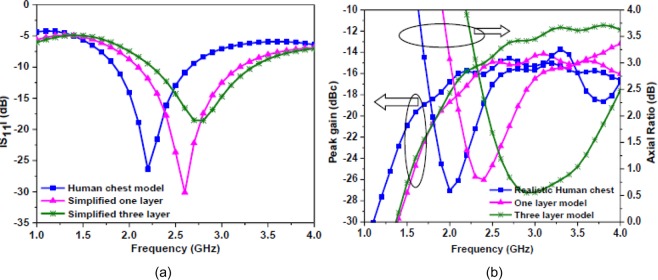
Performance comparison in homogeneous and heterogeneous environments (**a**) simulated |S_11_ | (**b**) simulated gain and axial ratio.

Radiation performance of the antenna in a homogeneous simplified one-layer skin model and heterogeneous environment of the human body is validated. The antenna is placed inside the vertical stack-based homogeneous simplified three-layer model (skin + fat + muscle)^32^ and heterogeneous realistic voxel-based model of the human body (AustinMan v 2.6) as shown in Fig. 13(a),(b), respectively. A radiation box of 600 × 400 × 800 mm^3^ shows the portion of the body considered during simulations. Realistic human body model has a voxel resolution of 1 × 1 × 1 mm^3^ with 104, 328, 722 voxels that have been developed from the National Library of Medicine’s Visible Human Project data set^33^. Note, during the simulations, the antenna is placed 4 mm below the top layer (skin) in the three-layer model and at 4 mm depth in the chest (muscles) in the realistic model. The electrical properties of the biological tissues are taken from^34–36^. Here, numerical analyses are conducted using CST Microwave Studio Suite, using CST (.vox + .lat) files. Figure 14(a),(b) shows |S_11_ | , gain and axial ratio plots of the antenna, respectively. From the graphs, a shift in resonant frequency and axial ratio band is seen, whereas the gain is not affected much. The change in resonant frequency could be due to the asymmetric environment of the human body which would have affected the impedance and polarization of the antenna. In the three-layer model, frequency shifts to the higher side as the antenna was implanted in the fat below the skin layer. Fat has a lower dielectric constant than skin. In the realistic human body model, resonant frequency shifts to a lower side due to the high dielectric constant of muscle tissue.

#### Influence of implantable depth (d)

**Figure 15:**
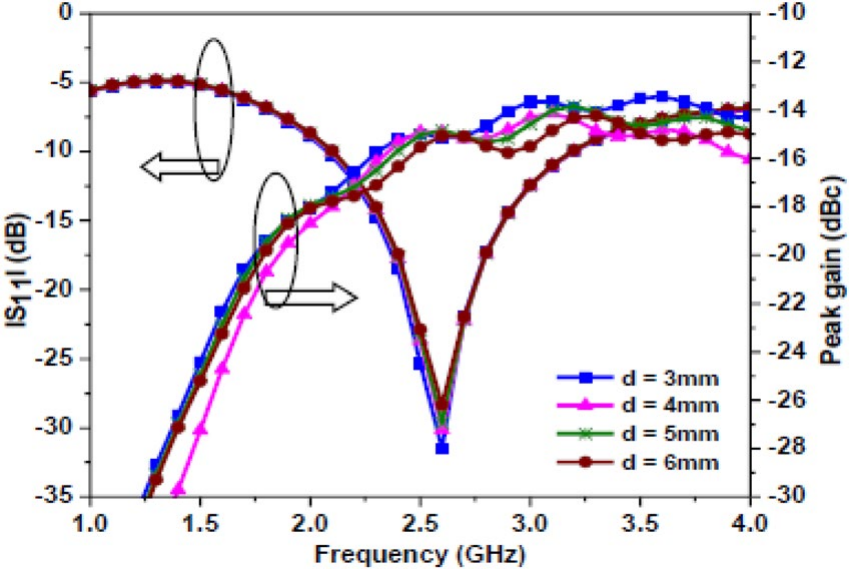
Simulated |S_11_ | and gain comparison at different implantable depths.

Initially, the proposed antenna is simulated in a homogeneous simplified one-layer skin tissue model at a depth of (*d*) 4 mm. In practice, the implantation depth could vary from case to case, which may lead to variation in antenna radiation performance. Therefore, the effect of depth is studied by varying its value in the skin tissue model. Figure 15 shows |S_11_ | and gain plots for the different values of *d* with an interval of 1 mm. It is noticed that the return loss and gain values are almost the same, therefore, the proposed antenna can provide acceptable performance at different implantable depths. Note, the axial ratio values are also the same, but not shown here for brevity.

#### Biocompatibility of the proposed antenna

**Figure 16:**
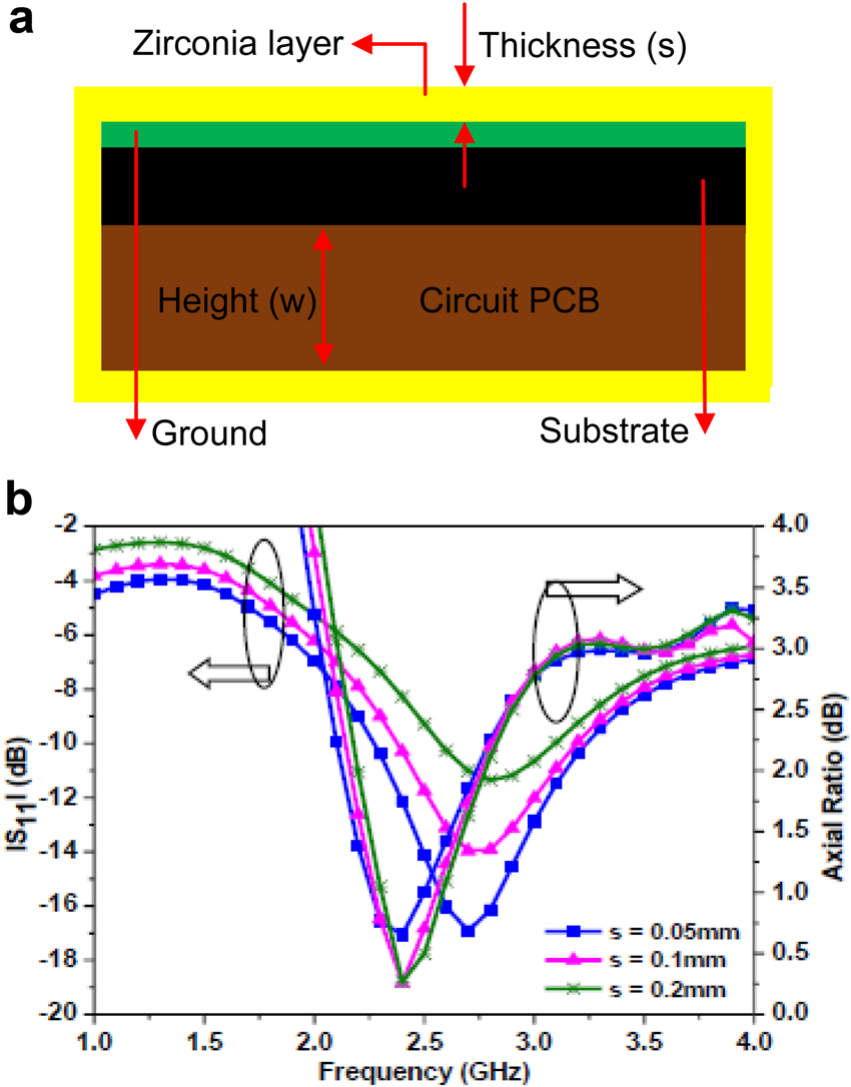
(**a**) Proposed antenna coated with biocompatible. layer. (**b**) Simulated |S_11_ | and axial ratio for different biocompatible layer thickness.

The proposed antenna has to carry out its functionality inside the human body and to avoid the effect of short circuit (variation in EM characteristics of the antenna due to high conducting nature of the human tissues with the conducting material), the antenna needs to be biocompatible. The biocompatibility of the proposed antenna can be analyzed by covering the antenna with a biocompatible layer. The entire surface of the proposed antenna is covered with a biocompatible layer of zirconia material (*ɛ*_*r*_ = 27, *σ* ≈ 0, tan *δ* ≈ 0) of thickness *s* = 0.05 mm as shown in Fig. 16(a). The antenna structure is simulated by using a simplified one-layer skin tissue model. In this study, the biocompatible layer of zirconia material is preferred due to its high dielectric constant as compared to other materials like Teflon, MACOR, ceramic alumina, PEEK, Silastic MDX-4210^2^. The performance of the proposed antenna is analysed by varying the thickness of the biocompatible layer. Figure 16(b) shows |S_11_ | and axial-ratio plots for different thickness of the biocompatible layer. It is noticed that as the thickness of the zirconia layer increases (from *s* = 0.05 mm), the |S_11_ | starts degrading and impedance bandwidth becomes narrow, but the axial ratio is not much affected. However, up to *s* = 0.2 mm, the proposed antenna provides acceptable performance. It is also noticed that the biocompatible layer enhances the gain of the proposed antenna up to −12 dBc for *s* = 0.2 mm.

**Figure 17:**
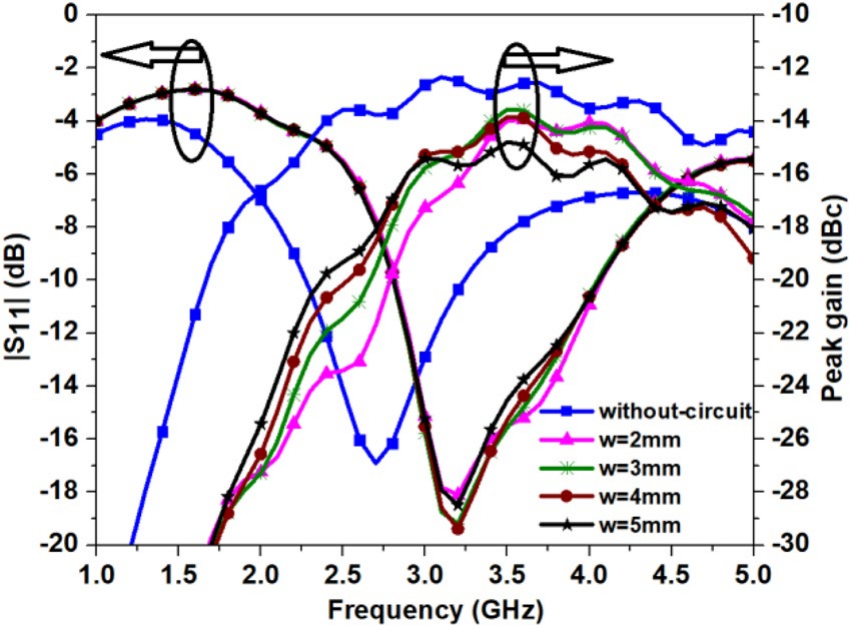
Simulated |S_11_ | and gain plot for different thickness of the PCB circuit.

Considering, the coupling between the proposed antenna and electrical components of the monitoring circuit (of the implantable devices), the robustness is analysed in the presence of the biocompatible layer (*s* = 0.05 mm). The electrical components are modelled as PEC cylinder (with radius = 5.2 mm and height (*w*) = 2 mm), characterizing circuit PCB underneath the substrate as shown in Fig. 16(a). Figure 17 shows |S_11_ | and gain curves for different thickness of the PCB by keeping *s* = 0.05 mm. When a PEC cylinder (*w* = 2 mm) is placed underneath the substrate, the |S_11_ | shifts to a higher frequency (3.2 GHz) and gain decreases to −23.4 dBc (at 2.45 GHz), as compared to the case (when *s* = 0.05 mm) without PEC cylinder. When the cylinder thickness increases up to *w* = 5 mm, the |S_11_ | values remain almost the same with no significant change in impedance bandwidth and resonant frequency, but gain value improves up to −19.3 dBc (at 2.45 GHz). It is also noticed that the axial ratio shifts to a lower frequency (2.2 GHz) and decreases to 2 dB for *w* = 2 mm, but it remains the same for a larger thickness of the cylinder. The results indicate that an acceptable radiation performance is obtained by covering the proposed antenna with a zirconia layer, however, the PEC cylinder affects the impedance and gain values somewhat. The detuning effect can be overcome by optimizing the dimensions of the proposed antenna.

#### Robustness of the proposed antenna in different tissues

**Figure 18:**
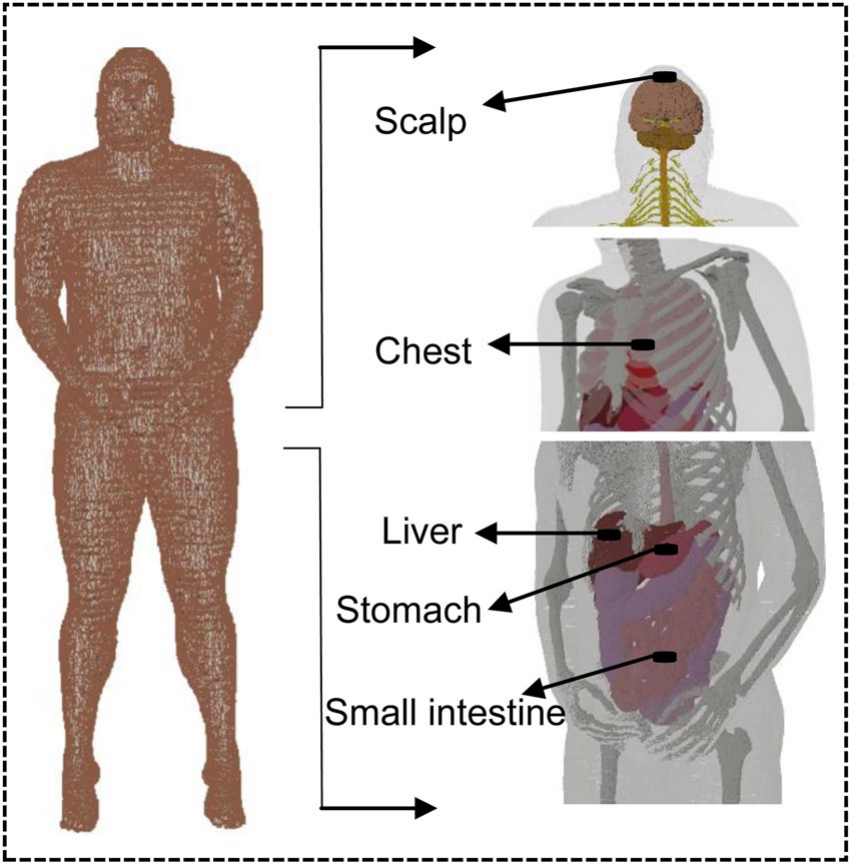
Antenna location in different parts of the body (of a realistic human body model).

**Figure 19:**
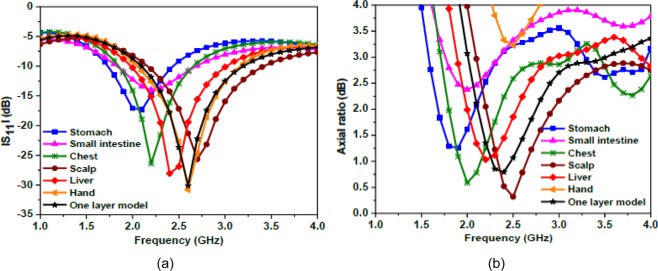
Performance comparison of the antenna for different body parts (**a**) simulated |S_11_ | (**b**) simulated axial ratio.

Furthermore, the performance of the proposed antenna is analyzed in various human tissues so that the antenna can be used for other biomedical applications also. For this, the proposed antenna is placed at a depth of 4 mm on different locations of the human body such as the stomach, small intestine, scalp, liver, and hand (of the realistic model of the human body) as shown in Fig. 18. Figure 19(a),(b) shows the respective |S_11_ | and axial ratio curves of the proposed antenna for the mentioned body parts. As compared to the simplified one-layer model, the |S_11_ | and axial ratio curves shift to lower frequency side due to stomach, and to higher frequency side due to scalp. In the case of scalp and hand, the antenna is located almost in the skin having the lowest permittivity value among other mentioned tissues. Stomach and small intestine also affect the impedance of the antenna considerably, due to their high permittivity values. On the other hand, the spherical and cylindrical shapes of the body parts do not affect the proposed antenna performance significantly.

## Measured and Simulated Results

### S_11_ and axial ratio measurement

**Figure 20:**
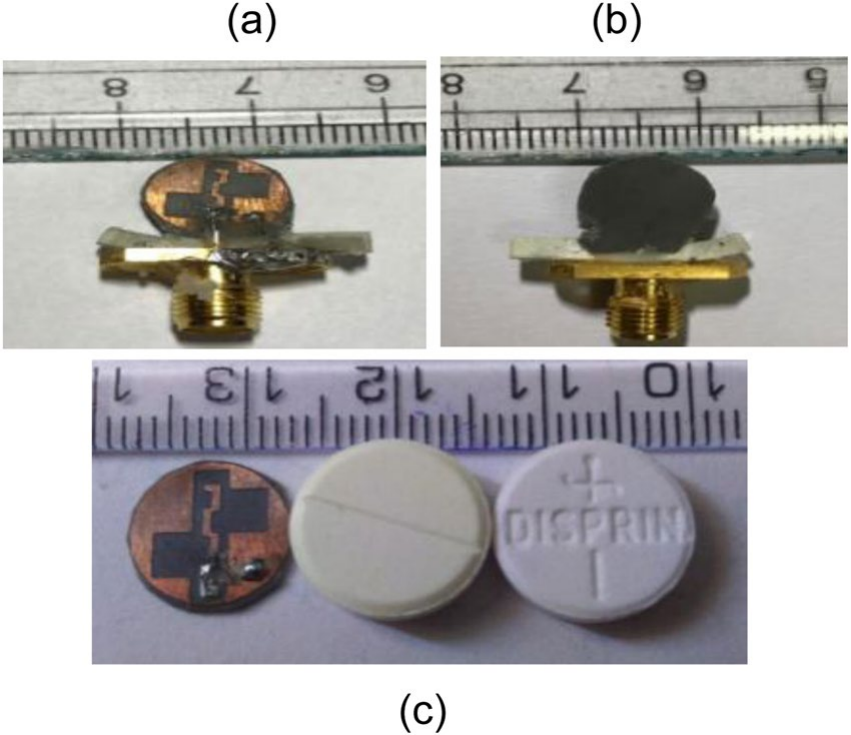
Fabricated antenna prototype (**a**) top view (**b**) bottom view (**c**) size comparison of the proposed antenna with medical tablets (pills).

**Figure 21:**
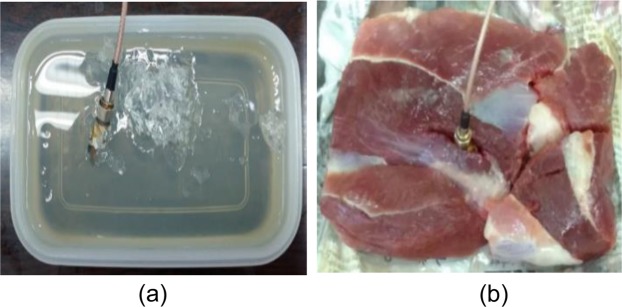
Measurement of the proposed antenna (**a**) human skin mimicking phantom (**b**) pork.

Figure 20 shows a fabricated prototype of the proposed ground radiating antenna, which conforms to the comparable antenna shape and size to a medical tablet (pill). The antenna performance is measured using Agilent N9914A PNA network analyzer *in vitro* and vivo conditions within a solid phantom and pork, respectively. For the measurements, the fabricated antenna was introduced in a rectangular plastic container and pork with the dimensions of 120 mm × 80 mm × 40 mm. The container is filled with the skin mimicking phantom^37–39^ prepared using the following steps: (1) firstly, sucrose (53%) and deionized water (47%) are added in a 100 ml beaker and stirred for about 20 minutes; (2) 1 g of dry agarose is added in 100 ml liquid solution; (3) a clear solution is obtained which is heated at ~80 °C for 1 hour; (4) the heated solution is left to cool at room temperature which get converted into a gel form. The proposed antenna is placed 4 mm below the top surface of the phantom and of pork to keep measurement procedure approximately in accordance with the simulated one (as shown in Fig. 21).

**Figure 22:**
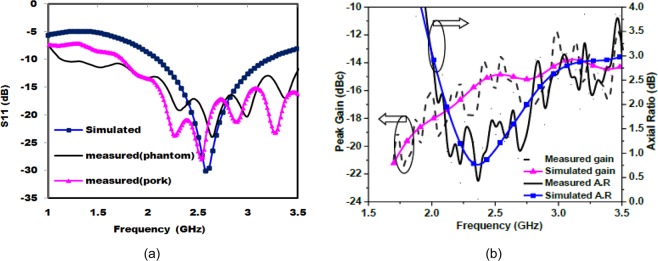
Simulated (one-layer skin model) and measured (**a**) |S_11_ | (**b**) gain and axial ratio.

The |S_11_ | is measured in both skin-mimicking phantom and pork while the axial ratio and radiation pattern measurements are carried out for phantom only. Figure 22(a) illustrates the comparison of simulated (in one-layer skin mimicking phantom model) and measured (in both phantom and pork) |S_11_ |. From the results, it is confirmed that the proposed antenna is exhibiting |S_11_ | below −15 dB for all the three cases (at 2.45 GHz design frequency). The simulated −10 dB impedance bandwidth is 1100 MHz (42.30%) from 2.1–3.2 GHz. The measured impedance bandwidth in phantom is 2680 MHz (99.25%) from 1.12–3.8 GHz. As can be seen, the measured |S_11_ | shows four resonances, whereas the simulated S_11_ shows a single resonance. The occurrence of multiple resonances, in the measured S_11_, was observed in the literature also^29,40–42^, and is due to the air gaps between the antenna and the medium of the phantom/pork, and cables and connector connected to the antenna prototype. Figure 22(b) represents simulated (one-layer skin model) and measured 3-dB axial ratio bandwidths of the proposed antenna. The simulated axial ratio bandwidth is around 1500 MHz (61.22%) from 2–3.5 GHz in the main radiation direction (*θ* = 0°). The measured axial ratio bandwidth in phantom is about 1080 MHz (44%) from 1.95–3.03 GHz in the direction *θ* = 0°. Compared with the simulated results, the measured results are slightly varying with a negligible shift at the resonant frequency. This effect is possibly due to the use of SMA connector and feeding coaxial cable in the measurement setup, where current is generated on the connector and cable’s surface due to the loading of lossy phantom material. Note, the lumped port is used during the simulation, which is similar to the scenario when the antenna is directly connected to the monitoring circuit output. As lumped port was not easily realizable during measurement, therefore, SMA connector was used for simplicity.

### Far-field measurement

**Figure 23:**
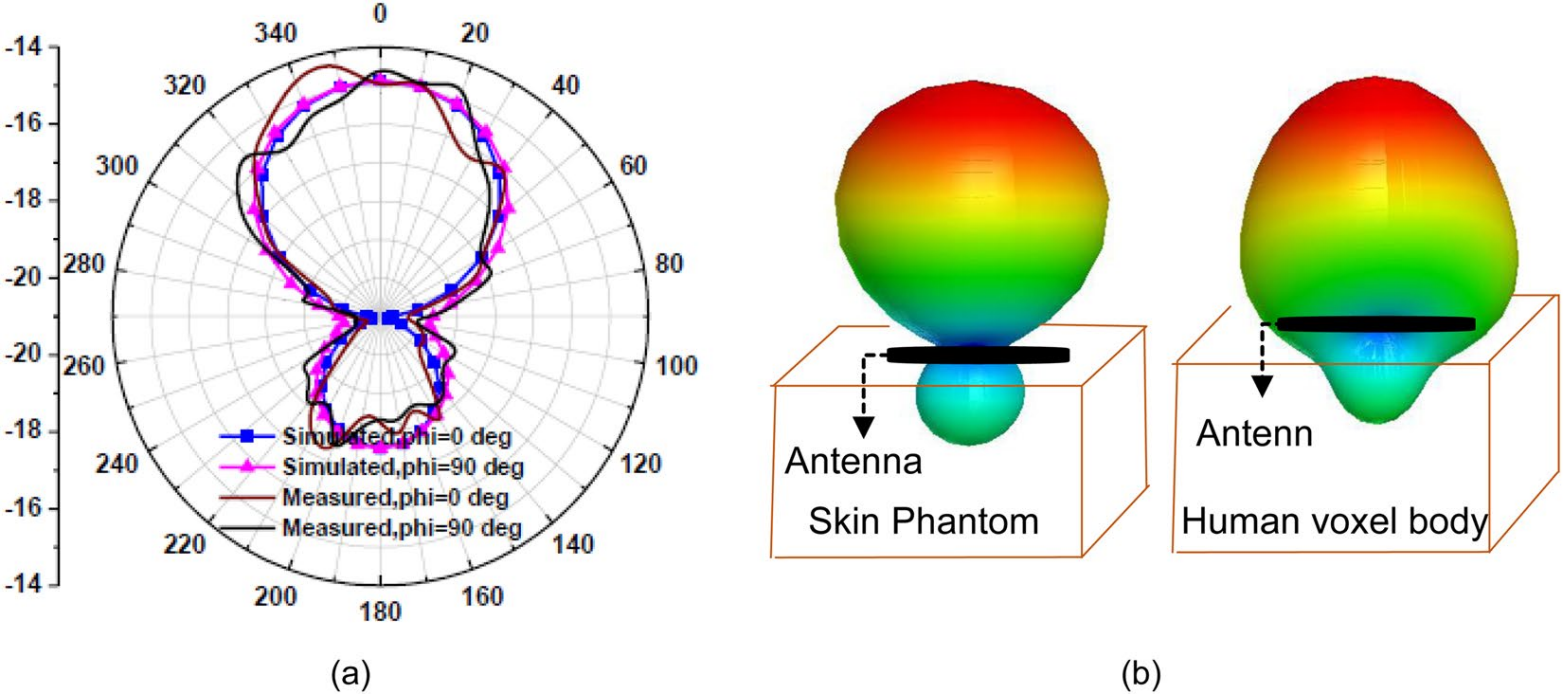
Radiation patterns at 2.45 GHz (**a**) 2-D (**b**) 3-D polar plots.

**Figure 24:**
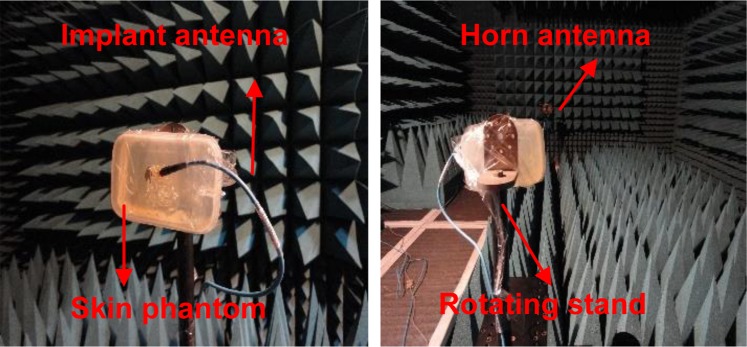
Far-field measurement setup inside an anechoic chamber.

The simulated and measured far-field broadside radiation patterns in homogeneous simplified one-layer skin tissue model at 2.45 GHz are shown in Fig. 23(a), which displays almost similar patterns in both E-plane and H-plane. Due to the realistic human body model (AustinMan) Fig. 23(b) shows insignificant distortion in radiation patterns as compared to the skin phantom. This distortion increases front lobe of the pattern in *z* > 0 direction and reduces back lobe in *z* < 0 direction. This reduction in back lobe eliminates the possibility of heating tissues from the back side of the antenna by reducing SAR. The proposed antenna has maximum RHCP radiation in *θ* = 0° direction. LHCP radiations are about 25 dB lower than the RHCP radiations in *θ* = 0° direction. Figure 22(b) represents simulated and measured peak gain of the antenna in the single-layer skin phantom. The simulated and measured gain around −15 dBc is obtained in *θ* = 0° direction. The far-field measurements were carried out inside an anechoic chamber in E-plane (*ϕ* = 0°) and H-plane (*ϕ* = 90°) by varying *θ* from 0° to 180° as shown in Fig. 24.

## Communication Link

### Link margin

To establish far-field communication link between the implanted antenna (transmitter, ${T}_{x}$) and external antenna (receiver, ${R}_{x}$), the communication link budget is calculated through the following equations^43^

2 $$LM\,(dB)=Link\frac{c}{{N}_{o}}(dB)-Required\,\frac{c}{{N}_{o}}(dB)$$

3 $$Link\frac{c}{{N}_{o}}(dB)=EIRP-{L}_{f}+{G}_{r}-{N}_{o}$$

4 $$EIRP\,(dB)={P}_{t}+{G}_{t}$$

5 $$Required\,\frac{c}{{N}_{o}}\,(dB)=\frac{{E}_{b}}{{N}_{o}}+10\,lo{g}_{10}({B}_{r})-{G}_{c}+{G}_{d}$$

where ${P}_{t}$ is the transmit power, ${G}_{t}$ is the transmitting antenna gain, ${G}_{r}$ is the receiving antenna gain, ${N}_{o}$ is the noise power density, *E*_*b*_ is the energy per bit and *B*_*r*_ is the bit rate. ${T}_{x}$ and ${R}_{x}$ antenna gains are based on simulated results and according to the free space reduction in signal strength, path loss in free space ${L}_{f}$ is calculated as

6 $${L}_{f}=20\,lo{g}_{10}\left(\frac{4\pi x}{\lambda }\right)dB$$

with distance (*x*) between ${T}_{x}$ antenna and ${R}_{x}$ antenna.

**Figure 25:**
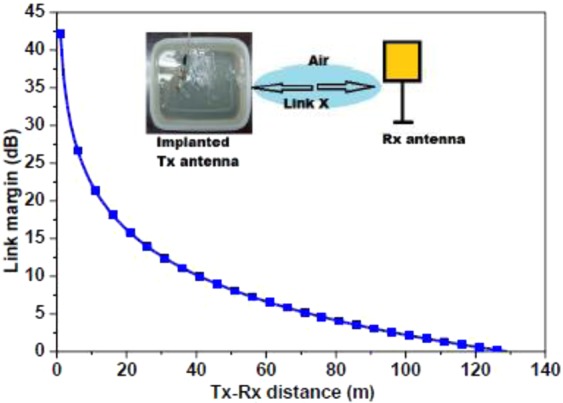
Simulated link margin plot between ${T}_{x}$ and ${R}_{x}$ antennas.

Here, ${T}_{x}$ and ${R}_{x}\,$polarization mismatch loss and impedance mismatch loss should also be included in the equation $(3)$, but both these losses are ignored in this study for simplicity. The link margin in Fig. 25 is calculated using the parameters given in Table 3. For effective communication, the link margin must be greater than 0 dB and it is observed that a communication between two antennas can be established effectively up to 129 m in the case of proposed antenna for a bit rate of 1 Mbps and input power of 100 µW. Since the data rate of 1–2 Mbps is sufficient to transmit data fast^43^, therefore, for a high data rate of 2 Mbps or more and ERC limit of 25 µW input power (sufficient for skin implantation), the wireless communication range will reduce. However, this reduction does not matter much as link margin of 15 dB is considered enough for practical applications^32^, which corresponds to more than 20 m range. The input power of 100 µW increases the communication range, but the EIRP (−25 dBm) of the proposed antenna should be maintained within the *EIRP*_*max*_ limits, where *EIRP*_*max*_ = 20 dBm at 2.45 GHz.

**Table 3:** Link budget parameters.

**Transmitter**	
Central Frequency (GHz)	2.45
Transmitted power, ${P}_{t}$ (dBW)	−40 (100 µW)
${T}_{x}$ antenna gain, ${G}_{t}$ (dBc)	−15.02
EIRP (dBW)	−55.02
**Receiver**	
${R}_{x}$ antenna gain, ${G}_{r}$ (dBi)	7.39
Temperature, ${T}_{o}$ (K)	293
Boltzmann constant, K	−1.38E-23
Noise power density, ${N}_{o}$ (dB/Hz)	−202.17
**Signal quality**	
Bit rate, ${B}_{r}$ (Mb/s)	1
Bit error rate	1E-5
${E}_{b}$/${N}_{o}$ (ideal PSK) (dB)	9.6
Coding gain, ${G}_{c}$ (dB)	0
Fixing deterioration, ${G}_{d}$ (dB)	2.5

### Specific absorption rate distribution

The EM-power absorbed by biological tissue (per unit) surrounding the proposed antenna has also been studied. IEEE C95.1–1999 (1 g-avg SAR < 1.6 W/Kg) and IEEE C95.1–2005 (10 g-avg SAR < 2 W/Kg) are the regulations restricting the input power of implantable antennas^44,45^. In a three-layer phantom model, the peak SAR values are 482.4 W/Kg and 31.15 W/Kg for 1 g and 10 g cubic tissue, respectively, at 2.45 GHz, when 1 W of input power is applied. Therefore, the net input power must be <3.32 mW and <64.20 mW for 1 g and 10 g cubic tissue, respectively to satisfy the IEEE guidelines. Since an input power of −40 dBW (100 µW) is much lesser than the transmitter power^43^, therefore, SAR is not a matter of concern for the proposed antenna.

## Conclusion

In this paper, a low profile CPW-fed CP ground radiating antenna operating at 2.45 GHz (ISM band) is proposed for wireless biomedical applications. The proposed miniaturized antenna exhibit broad impedance bandwidth and axial ratio bandwidth compared to other reported structures. Numerical simulations are carried out along with parametric studies and it is found that the measured results of the antenna are in agreement with the simulations. The antenna is robust to different tissue exposure. The link margin of the antenna is also calculated to demonstrate its wireless communication ability. Additionally, SAR distribution is discussed which is also in the acceptable range. Hence, the proposed antenna is very much compact, economical, easy to fabricate, highly efficient and will be covering very less area on the IMDs.
